# Selective JAK-Inhibitors in Spondyloarthritis

**DOI:** 10.31138/mjr.311023.sji

**Published:** 2024-03-30

**Authors:** Konstantinos D. Vassilakis, Konstantina Magiouf, Stefan Siebert, George E. Fragoulis

**Affiliations:** 1 Joint Academic Rheumatology Program, First Department of Propaedeutic and Internal Medicine, National and Kapodistrian University of Athens, Athens, Greece,; 2 School of Infection and Immunity, University of Glasgow, United Kingdom

**Keywords:** JAK-inhibitors, spondylarthritis, PsA, axSpA, efficacy, safety

## Abstract

As our research interest and knowledge increases in the field of Spondyloarthritis, new aspects also emerge as regards to their therapeutic approach. JAK inhibitors (JAKi) are a relatively new treatment option, aiming molecules in the JAK-STAT pathway, which has a leading role in the pathophysiology of both Psoriatic Arthritis and Axial Spondyloarthritis. JAKi exhibit different selectivity towards the four different members of the JAK family (namely JAK1, JAK2, JAK3, and TYK2), possibly reflecting different efficacy and safety profile. Although knowledge is more consolidated for rheumatoid arthritis in which JAKi are being used for more than 10 years, data are still accumulating for PsA/SpA. In this review we aim to present and assess current knowledge about the efficacy of JAKi (with a focus on selective JAKi) in the treatment of patients with SpA and evaluate their safety profile as some concerns may arise around this therapeutic option.

## INTRODUCTION

Spondyloarthritis (SpA) comprises a group of related clinical entities characterised primarily by inflammation of the spine and joints, in addition to sharing a variety of other clinical manifestations. Among the various SpA subtypes, psoriatic arthritis (PsA) and axial spondyloarthritis (axSpA) are the two most common.

PsA is mainly characterised by inflammatory arthritis, with axial involvement being present in about 20–50% of PsA patients.^1^ Psoriasis is almost always present, while dactylitis, enthesitis, and nail disease are also commonly observed. On the other hand, axSpA typically affects the axial skeleton, manifested by inflammatory back pain and stiffness, and is further divided into non-radiographic axSpA (nr-axSpA) and radiographic axSpA (r-axSpA, previously known as ankylosing spondylitis), based on radiographic findings. Extra-musculoskeletal manifestations such as uveitis and inflammatory bowel disease (IBD) can be seen in both PsA and axSpA, although they are more common in the latter. Furthermore, comorbidities such as cardiovascular disease and mental health disorders are increasingly recognised as important features of these diseases.^2,3^

Approved therapeutic options in PsA include conventional disease modifying antirheumatic drugs (DMARDs), biologic DMARDs (bDMARDs) and targeted synthetic DMARDs (tsDMARDs) [JAK-inhibitors (JAKi) and apremilast], whereas the current licensed drug treatments in ax-SpA mainly include b- and ts-DMARDs.^4,5^ JAK inhibitors have been recently introduced as a treatment strategy across a range of inflammatory rheumatic conditions.^6^ A number of JAKi are now in phase-3 trials or already licensed for PsA and axSpA (**Table 1**). Although, the various JAKi belong to the same family, there are differences regarding which JAK members that are inhibited, reflected in their efficacy and possibly also their safety. In fact, in contrast to the other members of the family, upadacitinib and filgotinib seem to be more “selective” affecting mainly JAK1.

**Table 1.** JAK inhibitors currently approved by the European Medicines Agency for adult rheumatic diseases.

**JAK Inhibitor**	**Selectivity**	**Diseases**						**First Approval in EMA**
		**RA**	**PsA**	**UC**	**nr-AxSpA**	**AS**	**CD**	
Tofacitinib	JAK 1,2,3	✓	✓	✓				2017
Baricitinib	JAK 1,2	✓						2017
Upadacitinib	JAK 1	✓	✓	✓	✓	✓	✓	2019
Filgotinib	JAK 1	✓		✓				2020

RA: Rheumatoid Arthritis; PsA: Psoriatic Arthritis; UC: Ulcerative Colitis; nr-AxSpA: non-radiographic Spondylarthritis; AS: Ankylosing Spondylitis; CD: Crohn’s Disease.

## JAK-INHIBITORS

Cytokines serve as crucial mediators in the inflammatory process, playing a central position in the development and perpetuation of a range of autoimmune and inflammatory conditions. Hence, cytokine inhibition is one of the central therapeutic strategies in the field of rheumatology.^7^ In recent years, there has been a growing interest in the effectiveness of small-molecule treatments that target crucial signal transmitters in the downstream pathways of multiple cytokines and other molecules, for the management of rheumatic and autoimmune disorders, with JAKi at the forefront of these.^8^

The JAK family of non-receptor tyrosine kinases includes four proteins: JAK1, JAK2, JAK3, and TYK2.^9^ These are cytoplasmic tyrosine kinases capable of phosphorylating tyrosine residues, either within their own structure (autophosphorylation) or on neighbouring molecules (transphosphorylation), including the STAT proteins.^10^ Among mammals, JAK1, JAK2, and TYK2 exhibit widespread expression throughout various tissues. In contrast, JAK3 expression is more limited, being primarily found in haematopoietic cells.^9^ JAK inhibitors selectively block ATP-binding sites of JAKs, dampening downstream signalling, and potentially modulating various immune responses, including those involved in rheumatologic diseases.^11^ Currently, there are four small molecule JAK inhibitors (JAKi) in use within the field of rheumatology (**Table 1**), each with varying degrees of selectivity for the four distinct JAK isoforms: tofacitinib, baricitinib, upadacitinib, and filgotinib, with the last two also being known as “selective-JAK inhibitors” as they block only JAK1.^12^ JAK inhibitors have been approved for the treatment of a variety of immune mediated conditions (**Table 1**) including various forms of inflammatory arthritis and IBD. Herein we review the selective JAK1 inhibitors upadacitinib and filgotinib in PsA and axSpA.

## EFFICACY

### Psoriatic arthritis

#### Upadacitinib

The efficacy of selective JAKi in PsA has been thus far examined in clinical trials. In a phase 3 long-term clinical trial (SELECT-PSA 1), the efficacy of upadacitinib was evaluated in PsA patients. Adult patients with active PsA [fulfilling Classification Criteria for Psoriatic Arthritis (CASPAR)] and an inadequate response or intolerance to one or more non-bDMARD were randomised in a 1:1:1:1 ratio to receive oral upadacitinib 15mg or 30mg once a day, placebo, or subcutaneous adalimumab 40mg every other week. Patients receiving active treatment continued to take the therapy throughout the trial, whereas patients assigned to placebo at baseline were switched to upadacitinib (15mg or 30mg once daily) at week 24. The primary outcome of American College of Rheumatology 20 (ACR20) response was achieved at week 12 by a significantly greater proportion of participants receiving upadacitinib [70.6% and 78.5% for 15 and 30mg, respectively] compared with those taking placebo [36.2%], while ACR20 responses up to week 24 were non-inferior for upadacitinib compared to individuals on adalimumab (65.0% at week 12). In fact, the group receiving upadacitinib 30mg every day, displayed significantly better ACR20 responses than those receiving adalimumab at both timepoints (78.5% vs 65.5% at week 12 and 78.5% vs 67.1% at week 24). Response was generally maintained throughout week 104. Secondary endpoints, such as improvement in Psoriasis Area and Severity Index (PASI75/90/100), achievement of minimal disease activity (MDA), and resolution of enthesitis and/or dactylitis were also achieved in more patients treated with upadacitinib than placebo at week 12 and were maintained up to week 104. It is worth mentioning that as regards to ACR50/70 achievement, upadacitinib at 15mg qd was superior to adalimumab in some (eg, weeks 24, 36, 56) of the time points assessed.^13–15^

In a study assessing efficacy of upadacitinib in patients who have failed at least one bDMARD (phase 3 SELECT-PSA 2 trial), 642 patients were randomised to receive therapy (2:2:1:1) with oral upadacitinib at a dose of 15 or 30mg or placebo, followed by switching to upadacitinib 15 or 30mg once a day at week 24. The outcomes were comparable with those of biologic-naïve patients seen in SELECT-PSA 1 with improvement across PsA domains. The proportion of patients achieving the primary outcome ACR20 at week 12 were 59.7% and 59.2% for upadacitinib 15mg and 30mg, respectively, compared 24.1% for placebo (p<0.001 for both comparisons). MDA at week 24 was achieved by greater percentages of patients receiving the active drug compared to placebo (upadacitinib 15mg: 25.1%, upadacitinib 30mg: 28.9% placebo: 2.8%, p<0.001 for both). Similarly, psoriasis, enthesitis, dactylitis and patient-reported outcomes were improved with upadacitinib at week 24, compared to placebo, and maintained through 56 weeks.^16^ Upadacitinib has now been approved for the treatment of PsA but real-world data are still relatively lacking thus far.

**Table 2.** Risk of opportunistic infections with JAK inhibitors.

**Author – Year**	**Type of study**	**ARD**	**JAK inhibitors**	**Comparator**	**Results**
***Opportunistic Infections (OI)***														
Choi - 2023^30^	Retrospective	RA	Tofa or Bari	TNFi	HR (95% CI): 0.25 (0.09–0.73)
Vassilopoulos - 2022^34^	Meta-analysis	PsA	Deucra, Tofa, Upa, Filgo	TNFi, anti-IL-17, anti-IL-23	^ JAKi: 2.72% (95% CI: 1.05%–5.04%), anti-IL-17: 1.18% (95% CI: 0.60%–1.9%), anti-IL-23: 0.24% (95% CI: 0.04%–0.54%), anti-TNFs: 0.01% (95% CI: 0.00%–0.21%)
***Infections***														
Choi - 2023^30^	Retrospective	RA	Tofa or Bari	TNFi	HR (95% CI): 1.04 (0.71–1.52)
Yang - 2023^35^	Meta-analysis	PsA	Tofa, Bari, Upa, Filgo, Peci, Solci, Abro and Deucra	Placebo	RR (95% CI): 1.20 (1.07–1.35)
Mok - 2023^33^	Retrospective	RA	JAKi	TNFi	HR (95% CI): 1.08 (0.84, 1.39)
Uchida - 2023^31^	Retrospective	RA	Tofa or Bari	TNFi	HR (95% CI): 0.79 (0.42–1.50)
***Herpes Zoster (HZ)***														
Choi - 2023^30^	Retrospective	RA	Tofa or Bari	TNFi¶	HR (95% CI): 2.37 (2.00–2.80)
Uchida - 2023^31^	Retrospective	RA	Tofa or Bari	TNFi	HR (95% CI): 0.20 (0.08–0.52)
Mok - 2023^33^	Retrospective	RA	JAKi	TNFi	3.49 vs 0.94 /100 py, p < 0.001
Xu - 2023^38^	Meta-analysis	IBD, RA, SpA, PsO, PsA	JAKi	placebo	OR (95% CI)^#^; Bari: 3.46 (1.38, 8.67), Pefi: 6.06, (1.76, 20.82), upa ¬ : 3.87, (1.07,13.98)
Redeker 2021^37^	Retrospective	RA	JAKi	csDMARDs	HR (95% CI): 3.23 (2.32–4.48)
***Malignancy***														
Russell - 2023^39^	Meta- analysis	RA, PsA, axSpA, IBD, AD	Tofa, Bari, Upa, Filgo, Pefi	placebo, TNFi or MTX	IRR (95% CI): 1.63(1.27–2.09) **
Huss - 2023^41^	Retrospective	RA or PsA	Bari, Tofa, Upa	bDMARDs	HR (95% CI): JAKi: 0.94 (0.65–1.38) (Vs TNFi)^¥^
Song - 2022^42^	Retrospective	RA	JAKi	TNFi	HR (95% CI): 0.83 (0.55–1.27)
Mok - 2023^33^	Retrospective	RA	JAKi	TNFi	HR (95% CI): 0.87 (0.39, 1.95)
Uchida - 2023^31^	Retrospective	RA	Tofa or Bari	TNFi or general population	HR (95% CI): 0.385 (0.095–1.552) (Vs TNFi)
Yang - 2023^35^	Meta-analysis	PsA	Tofa, Bari, Upa, Filgo, Peci, Solci, Abro and Deucra	Placebo	RR (95% CI): 2.20 (0.83–5.86)
***Cardiovascular Events (CVDs)***															
Mok - 2023^33^	Retrospective	RA	JAKi	TNFi	HR (95% CI): 1.36 (0.62, 2.96)
Yang - 2023^35^	Meta-analysis	PsA	Tofa, Bari, Upa, Filgo, Peci, Solci, Abro, and Deucra	Placebo	RR (95% CI): 1.18 (0.43–3.20)	
Hoisnard 2022^52^		RA	Tofa, Bari	ADA	HR (95% CI): 1.0 (0.7–1.5)
Khosrow-Khavar - 2022^47^	Retrospective	RA	Tofa	TNFi	Composite CV outcome - HR (95% CI): 1.01 (0.83–1.23)
Kremer - 2021^53^	Retrospective	RA	Tofa	bDMARDs	HR (95% CI): 0.61 (0.34–1.06)
Salinas - 2022^54^	Retrospective	RA	Bari	TNFi	IRR (95% CI): 1.54 (0.93–2.54)
Ytterberg 2022^44^	Prospective	RA	Tofa	TNFi (ADA, ETC)	HR (95% CI): 1.24 (0.81–1.91)
***Thromboembolic Events (VTEs)***															
Song - 2023^50^	Retrospective	RA	JAKi	TNFi	HR (95% CI): 0.18 (0.01–3.47)
Desai - 2022^55^		RA	Tofa	TNFi	HR (95% CI): 1.13 (0.77–1.65)
Hoisnard 2022^52^	Retrospective	RA	Tofa, Bari	ADA	HR (95% CI): 1.1 (0.7–1.6)
Molander -2022^49^	Retrospective	RA	Tofa, Bari	TNFi or non-anti-TNF bDMARDs	HR (95% CI): VTE: 1.73 (1.24–2.42), PE: 3.21 (2.11–4.88), DVT: 0.83 (0.47–1.45)
Salinas - 2022^54^	Retrospective	RA	Bari	TNFi	IRR: 1.51 (1.10–2.08)
Ytterberg 2022^44^	Prospective	RA	Tofa	TNFi (ADA, ETC)	HR (95% CI): 1.66 (0.76–3.63)
Yang - 2023^35^	Meta-analysis	PsA (with PsO)	Tofa, Bari, Upa, Filgo, Peci, Solci, Abro, and Deucra	Placebo	RR (95% CI): 0.83 (0.11–6.24)

ARD: Autoimmune Rheumatic Disease; Tx: Treatment; RA: Rheumatoid Arthritis; PsA: Psoriatic Arthritis; PsO: Psoriasis; axSpA: Axial Spondyloarthritis; AS: Ankylosing Spondylitis; IBD: Inflammatory Bowel Disease; or UC: Ulcerative Colitis; AD: Atopic Dermatitis; JAKi: JAK inhibitors; MTX: Methotrexate; GC: Glucocorticoids; PRD: Prendisolone; TNFi: TNF inhibitors; bDMARDs: biologic DMARDs; csDMARDs: conventional synthetic DMARDs; EAIR: Exposure-adjusted incidence rate; PY: Person-Years; QD: quaque di i.e. once a day; NMSC: Non Melanoma Skin Cancer; MACE: Major Adverse Cardiovascular Events; Tofa: Tofacitinib; Bari: Baricitinib; Deucra: Deucravacitinib; Upa: Upadacitinib; Filgo: Filgotinib; Pefi: Peficitinib; Solci: Solcitinib; Abro: Abrocitinib; INX: Infliximab; Ada: Adalimumab; Gol: Golimumab; ETN: Etanercept; HR: Hazard ratio; ITT: Intention-to-treat; RR: Relative risk; py: patients-years.

^ cumulative incidence risk; higher risk for JAK-inhibitors was attributed to the higher risk for herpes zoster

¶ Infiliximab, Adalimumab, Golimumab, Etanercept

# for RA patients,

¬ for 30mg/QD

* HR for serious HZ is also available

** Vs TNF; when ORAL Surveillance excluded the (IRR) Incidence Rate Ratio (95%CI): 0.71 (0.44–1.15)

¥ Higher risk (vs TNFi) for NMSC in RA [HR (95% CI): 1.39 (1.01–1.91)]

#### Filgotinib

In the EQUATOR multicentre, double-blind, placebo-controlled phase 2 trial, the efficacy of filgotinib was assessed in patients with PsA (fulfilling the CASPAR criteria). Individuals with moderate-to-severe disease [at least five swollen joints (from a 66 swollen joint count) and at least five tender joints (from a 68 tender joint count)], active or a documented history of plaque psoriasis and an insufficient response or intolerance to at least one csDMARD were enrolled. Participants (n=131) were randomised (1:1) to receive filgotinib 200mg or placebo once a day over a period of 16 weeks. The proportion of patients achieving ACR20 at week 16, the primary endpoint of the study, was greater for the active drug recipients. Specifically, 80% of the patients on the filgotinib arm and 33% of the placebo patients achieved an ACR20 response at week 16, with 47% treatment difference between the two groups (95% CI 30.2–59.6, p<0.0001). Secondary and exploratory endpoints were also assessed. ACR50 was attained from more patients on active drug than with placebo [treatment difference: 33% (95% CI 16.8–46.2]; p<0.0001)], as was ACR70 [treatment difference: 17% (4.9–29.2), p=0.0037]. Furthermore, at week 16, the musculoskeletal composite outcome Disease Activity Index for Psoriatic Arthritis (DAPSA) was also improved in patients treated with filgotinib compared to those who received placebo (least square [LS] mean difference −12·5 [95% CI −17.0 to −8.0, p<0.0001]. Moreover, more patients in the filgotinib group achieved MDA at week 16 with a treatment difference of 14% (p=0.0212). Additionally, filgotinib treatment was associated with improvement in psoriasis with a greater proportion of patients receiving filgotinib achieving PASI75 improvement compared with placebo, with a 30% treatment difference (p=0.0034). Filgotinib also resulted in more improvement of enthesitis compared to placebo, leading to improvement of SPARCC- [LS mean difference −1.4 (95% CI −2.6 to −0.1), p=0.0310] and Leeds- [LS mean difference −1.1 (95% CI −1.7 to −0.5), p=0.0004] enthesitis index. Lastly, multiple patient-reported outcomes, such as physical functioning, pain, and fatigue were also improved.^17^

### Axial Spondyloarthritis

#### Upadacitinib

The efficacy of upadacitinib in axSpA was evaluated in SELECT-AXIS 1, a multicentre, double-blind phase 2/3 study.^18–20^ Patients with active ankylosing spondylitis (as per the modified New York criteria) and an inadequate response to at least two NSAIDs (or intolerance to or contraindication for) were enrolled. Individuals (n=187) were randomly assigned (1:1) to upadacitinib 15mg or to placebo once daily over a period of 14 weeks (period 1). Patients completing period 1, were then eligible to participate in period 2 of the trial, 90-week open label extension, in which the long-term efficacy of upadacitinib was assessed with the patients in the placebo arm switched to active drug at week 14. The primary endpoint was the Assessment of SpondyloArthritis International Society 40 (ASAS40) response. At week 14, significantly greater proportion of patients in the upadacitinib group (52%) achieved an ASAS40 response, compared to the placebo arm (26%), with a treatment difference of 26% [p=0.0003; (95% CI 13–40)]. The mean change from baseline in Ankylosing Spondylitis Disease Activity Score (ASDAS) was greater in the active drug recipients −1.45 (95% CI −1.62 to −1.28) versus placebo −0.54 (95% CI −0.71 to −0.37). The Bath Ankylosing Spondylitis Functional Index (BASFI) improvement was also greater in the upadacitinib arm with a treatment difference of −1·00 [−1·60 to −0·39; p=0.0013]), while Bath Ankylosing Spondylitis Disease Activity Index 50 (BASDAI50) improvement was achieved from more patients in the upadacitinib group compared to placebo study sample (45% and 23%, respectively, with a treatment difference of 22%, p=0·0016). In addition to clinical measures, upadacitinib also improved the Spondyloarthritis Research Consortium of Canada (SPARCC) MRI sacroiliac joint score, a key secondary endpoint [change from baseline: −3.91 (−5.05 to −2.77)] versus placebo [−0.22 (−1.47 to 1.04), p<0.0001]. In the follow-up period beyond week 14, the ASAS40 response continued to increase in the continuous active drug group through weeks 32–40, at which point the responses started to plateau and were maintained through week 104 [85.9% of patients achieved ASAS40 at week 14; (95% CI 77.8 to 94). Secondary endpoints were also improved and were sustained through week 104.^18–20^

In another randomised, double-blind phase 3 study (SELECT-AXIS 2), the efficacy of upadacitinib was assessed in patients with non-radiographic axSpA. Adults with active non-radiographic axial spondylarthritis and an inadequate response to at least two NSAIDs or intolerance to or contraindication for NSAIDs were included in this trial. Patients were randomised (1:1) to receive upadacitinib 15mg daily or matched placebo over a period of 14 weeks. At week 14, the proportion of patients with an ASAS40 response (primary endpoint) was significantly greater for the upadacitinib recipients (45%) compared to placebo (23%) with a treatment difference of 22% (95% CI 12–32; p<0.0001). Secondary endpoints were also mostly met. Greater improvement from baseline in ASDAS was observed with upadacitinib with a mean change −1.36 compared to −0.71 on the placebo arm (p<0.0001), with more decrease of inflammation evident on MRI sacroiliac joint imaging as measured by SPARCC (−2.49 vs 0.57 for upadacitinib and placebo, respectively; p<0.0001). In addition, more patients treated with upadacitinib achieved BASDAI50 improvement compared to placebo (42% vs 22%; p=0.0001), with the former group also attaining a greater change in BASFI [p<0·0001]. Lastly, upadacitinib significantly improved patients’ quality of live as assessed by Ankylosing Spondylitis Quality of Life Questionnaire (ASQoL) [upadacitinib: −5.38 vs placebo: −3.15, p<0.0001] and ASAS Health Index [upadacitinib: −3.26 vs placebo: −1.48 p<0.0001].^21^ Upadacitinib has now been approved for the treatment of axSpA (including nr-axSpA) but real-world data are still relatively lacking thus far.

#### Filgotinib

In TORTUGA, a phase 2 trial, the efficacy of filgotinib was assessed in patients with active ankylosing spondylitis (modified New York criteria). Adults with active ankylosing spondylitis and an inadequate response to a at least two NSAIDs were randomly assigned to receive filgotinib 200mg once daily or placebo. At week 12, ASDAS change (primary endpoint) was significantly greater in patients receiving filgotinib −1.47 (standard deviation [SD]: 1.04) compared to those on placebo arm −0.57 (SD: 0.82), (p<0.0001). Secondary end points were also evaluated at week 12. The proportion of patients achieving an ASAS20 response was significantly greater for the filgotinib recipients (76%) compared to the placebo group (40%) (p<0.0001). BASDAI score displayed a greater decrease in the filgotinib group [mean change from baseline −2.41 (SD 2.01)] versus placebo [−1.44 (2.02)], p=0.0052), whereas BASFI was also reduced significantly more in the filgotinib arm [−2.45 (SD 1.90) vs −1.23 (1.88), p=0.0015]. Of note, a higher proportion of patients on the filgotinib arm [66% (27/41)] compared to placebo [18% (6/34)] had normal high-sensitivity CRP concentrations at week 12 with a difference of 48% (p<0.0001), while the change from baseline in CRP lab value was −10.84 mg/L (SD 13.91) with the JAKi and −2.24 mg/L (17.35) with placebo (p<0.0001). Filgotinib also improved the quality of life of individuals living with axSpA, with mean ASQoL score change of −4.76 (SD 4.50) in the active drug group versus −2.24 (3.97) for placebo (p=0.0038). In general, filgotinib was more efficient in reducing disease activity and signs and symptoms of active ankylosing spondylitis than placebo.^22^

### Safety

As there are now several effective therapeutic modalities available for the treatment of PsA and axSpA, safety regarding comorbidities, side effects, and adverse drug reactions is a critical consideration for physicians when selecting a treatment.

bDMARDs, including TNF, IL-17, and IL-23 inhibitors, have been used for many years in SpA with an acceptable and known safety profile. These drugs have been shown to carry no significant increased risk for malignancies, except non-melanoma skin cancers (NMSC).^23^ These bDMARDs are associated with slightly increased risk for infections, typically bacterial for TNFi and candidal infections for IL-17 inhibitors.^24,25^ Finally, it is important to note that these therapeutic modalities are associated with reduced cardiovascular diseases (CVD), a common comorbidity in both RA and PsA/axSpA.^26^

Most notably, in RA, TNF inhibitors have been shown to be protective for cardiovascular events (CVE) and Major Adverse Cardiovascular Events (MACE),^26^ whereas similar outcomes have been hypothesized for PsA based on data derived from psoriasis studies.^26–28^ The aspects above need to be considered when assessing the safety profile of JAKi, while the risk for Herpes Zoster Virus (HZV) reactivation merits special attention.

### Opportunistic infections

The risk of infection associated with JAK inhibitors constitutes an important point of interest and has been studied mostly in the setting of RA, with more long-term safety studies regarding their use in PsA and axSpA awaited. Data are more robust for older JAKinibs like tofacitinib and baricitinib and fewer for upadacitinib and filgotinib. In a structured literature review, opportunistic infections associated with JAKi for treatment in patients with RA were evaluated.^29^ Data and rates of opportunistic infections from 105 publications referring to 62 unique clinical trials were included. The most commonly reported opportunistic infection was reactivation of HZV leading to Zoster, whereas other opportunistic infections such as tuberculosis (TB), Pneumocystis Pneumonia (PCP), and candidiasis were reported more rarely. The crude frequency rate of TB did not exceed 0.5% for any JAK inhibitor at any timepoint (during the safety reporting period), with rates being higher in the Asian and Australian populations. Other rare opportunistic infections with a frequency of ≤1% included CMV (0.3%) reported with tofacitinib 5mg twice daily (BD), and aspergillosis (0.15%) and listeria monocytogenes infection (0.15%) reported with upadacitinib 15mg OD.^29^

In another population-based cohort study in Korea the infection risk of JAK inhibitors versus TNF inhibitors among RA patients was compared. The risk of severe bacterial infection was similar between the two drug categories, whereas the risk of opportunistic infection was significantly lower among JAKi vs TNF recipients.^30^ Along the same lines, in a retrospective study from Japan, risks for serious infections were similar between RA patients treated with JAK inhibitors or TNFi.^31^ Furthermore, a study examined the frequency of serious infections in RA patients participating in phase 2/3/4 clinical trials treated with tofacitinib or TNFi, as well as data from the US Corona registry, showed that incidence rates for all infections as well as for non-fatal serious infection events were similar for patients receiving tofacitinib 5mg BD or TNFi, irrespective of age.^32^ In contrast, a retrospective analysis of real-world data using the Hong Kong Biologics Registry to assess JAKi versus anti-TNFs safety in individuals with RA reported that the incidence of all infections was higher in JAKi than TNFi recipients after adjustment for age, sex, and disease duration (16.3 vs 9.9 per 100 person years (PY), p=0.02). These events were mostly genitourinary, lower respiratory tract and skin or soft tissue infections, although rates of severe infections (defined as those requiring hospitalisation) did not differ significantly between the two drug classes [JAKi: 8.2 - TNF:5.9 (per 100PY), p=0.77].^33^

A systematic review and meta-analysis published in 2022^34^ assessed the incidence of opportunistic infections in patients with PsA treated with biologic and targeted synthetic agents. For JAKi, the cumulative incidence of opportunistic infections was 2.72% (95% CI: 1.05%–5.04%), which was higher when indirectly compared to those for other bDMARDs [TNFi: 0.01%, (95% CI: 0.00%–0.21%), anti-IL-17: 1.18% (95% CI: 0.60%–1.9%), anti-IL-23: 0.24% (95% CI: 0.04%–0.54%)]. This difference is probably attributed to the higher incidence of HZV infection, which was most common type of opportunistic infection reported in patients treated with JAKi [cumulative incidence: 2.53% (95% CI: 1.03%–4.57%)].^34^ Lastly, a recent meta-analysis reported the safety of JAKi in patients with psoriasis and PsA. Among the 17 clinical trials included (n=6802 patients), infection was the most prevalent adverse effect.^35^ Compared to the placebo group, the risk of bacterial infection was higher in the treatment group (*RR* 1.20, 95% CI 1.07–1.35, *P* = 0.002). This was most pronounced for upper respiratory tract infections (*RR* 1.36, 95% CI 1.05–1.76, *P* = 0.02).^35^ Compared to placebo, relative risk for herpes zoster infection in patients with PsA treated with JAKi was 2.21 (95% CI 1.09–4.49, p = 0.03). In conclusion, apart from zoster, the risk of serious infections with JAKi does not appear significantly greater than TNFi in RA, with more long-term data required for PsA/SpA.

### Herpes Zoster

Herpes Zoster infection is a recognised adverse event associated with JAKi treatment, with higher incidence of HZV infection with the approved JAKinibs than either methotrexate (MTX) or TNFi.^36^

In a recent structured literature review in RA, it was estimated that the overall exposure-adjusted incidence rate (EAIR) [per 100PY] for any form of HZ infection ranged from 1.1 to 12.3 per 100 PY for approved doses of all JAKis.^29^ The EAIR was 12.3 for upadacitinib 15mg OD, 1.3 for patients treated with filgotinib 200mg OD with methotrexate and 1.5 in those receiving filgotinib monotherapy.^29^ Along the same lines, data from the German RABBIT registry reported higher risk for HZ for JAKi and TNFi, compared to csDMARDs (HR: 1.66 and 1.63, respectively).^37^ A cohort retrospective study in an Asian population reported that the EAIR of HZ for JAKi was nearly double that for TNFi (11.54 and 4.88 per 100 PY, respectively)^30^ while, a Japanese cohort study also noted an increased risk of HZ with JAKi compared with TNFi [TNFi vs JAKi HR for HZ: 0.200 (95% CI: 0.077, 0.524) p=0.001].^31^ Finally, real world evidence in RA from the Hong Kong Biologics Registry, estimated that the incidence of HZ is significantly higher with JAKi versus anti-TNFs (3.49 vs 0.94 per 100PY, p<0.001).^33^ Interestingly, in a recent meta-analysis, the higher risk of HZ infection in RA patients was evident only for RA patients treated with baricitinib 4 mg QD (OR = 3.46, 95%CI 1.38, 8.67), peficitinib 100 mg QD (OR = 6.06, 95%CI 1.76, 20.82), and upadacitinib 30 mg QD (OR = 3.87, 95%CI 1.07,13.98) versus placebo, but not for other JAKi (filgotinib, tofacitinib, ivarmacitinib, decernotinib) or other doses.^38^

Data for PsA and SpA are more limited. In a recent systematic review of clinical trials and real-world studies, the IR (/100 PY) and/or the cumulative incidence of HZ was evaluated in patients with a range of IMIDs, such as RA, PsA, ankylosing spondylitis, and ulcerative colitis, treated with tofacitinib, baricitinib or upadacitinib.^36^ Outcomes supported that the HZ incidence was higher in tofacitinib-treated individuals with RA (2.2–7.1 per 100 PY) or ulcerative colitis (1.3–7.6 per 100 PY) compared to PsA (1.7 per 100 PY). Regarding the latter, tofacitinib displayed a HZ-incidence between 0–3.3% in randomised controlled trials (RCTs), whereas incidence with the selective JAKi upadacitinib ranged between 0.9–1.4%.^36^ However, data from two different meta-analyses are contradictory. In the first one, examining data from 17 clinical trials (n=6802 patients) risk for HZ with JAKi was increased compared to placebo (relative risk (RR) 2.21; 95% CI 1.09–4.49, p = 0.03) in patients with psoriasis or PsA.^35^ In the second, data from 47 RCTs across different IMIDs were included. In subgroup analyses per disease and JAKi used, risk of HZ was not found to be increased with JAKi in patients with SpA or PsA, compared to placebo.^38^

In conclusion, it seems that in RA, JAKi confer an increased risk for HZ, with possible differences between JAKi, while more data are required to conclude whether the same applies for PsA and SpA.

### Malignancy

Another important aspect is whether an association exists between JAKi and neoplasia risk. A meta-analysis evaluating the risk of malignancy among patients with inflammatory joint (RA, PsA, axSpA), skin, and bowel diseases, reported a higher incidence of all malignancies including NMSC associated with JAKi compared with TNFi (incidence rate ratio (IRR) 1.50; 95% CI 1.16–1.94), but not in comparison with placebo (IRR 0.71; 95%CI 0.44–1.15) or methotrexate (IRR 0.77; 95% CI 0.35–1.68).^39^ It is important to note that this correlation of JAKi vs TNFi was primarily driven by the ORAL Surveillance trial, a large phase 4 safety study, which randomised patients with RA over 50 years of age and with additional cardiovascular risk factors to the JAKi tofacitinib or to a TNFi.^40^ When excluding this study, although no longer statistically significant, the effect estimates remain in the direction of higher malignancy IR with JAKi versus TNFi. Nevertheless, it is noteworthy, that malignancies were rare events across treatment groups, with an overall IR of 1 event per 100 PY of exposure.^39^ In contrast, a cohort study assessing cancer risks with JAKi compared to bDMARDs in patients with RA or PsA, indicated no evidence of increased short-term risk of all malignancies other than NMSC for JAKi versus bDMARD.^41^ It should be noted that in this study increased risk for NMSC in RA [HR: 1.39 (95% CI 1.01–1.91)] but not in PsA [HR: 2.05 (95% CI 0.79–5.31)] was described with JAKi.^41^ In a separate population-based study from Korea, overall risk of cancer (including solid cancers and haematological malignancies, analysed separately) was similar in RA patients treated with JAKi or TNFi.^42^ The Hong Kong Biologics Registry also reported no significantly different malignancy rates between JAKi or TNFi in RA patients (0.81 and 0.85 per 100 PY respectively; P= 0.25).^33^ Similarly, a retrospective study in Japan showed the malignancy standardised IR (SIR) of JAKi-treated patients was similar to those of the general population and of TNF recipients.^31^ Lastly, neoplasia risk of JAKi was also evaluated in a meta-analysis of RCTs including patients with psoriasis and PsA, with the overall incidence of malignancies in JAKi recipients similar to that in individuals in the placebo arm (*RR* 2.20, 95% CI 0.83–5.86, *P* = 0.11).^35^

### Cardiovascular and thromboembolic events

It is increasingly recognised that immune-mediated rheumatic diseases are characterized by an increased cardiovascular (CV) risk and CV mortality, when compared to the general population.^2^ The majority of the CV risk evidence relates to RA, with less evidence for SpA and PsA, despite worse metabolic profile in the latter.^2^ Thus, it is necessary to examine the effect of JAKi regarding to CVD, and more specifically MACE (stroke, myocardial infarction, CV death) and thromboembolic events (VTEs), including pulmonary embolism (PE) and deep vein thrombosis (DVT).

In RA, there appears to be a signal for association between JAKinibs and thromboembolic events, although more data are needed.^43^ The ORAL Surveillance trial was specifically designed to evaluate this in patients with RA aged over 50 years and with at least one additional CV risk factor, failed to demonstrate non-inferiority of tofacitinib, compared to TNFi, in regard to MACE endpoints.^44^ Post hoc analysis of the ORAL Surveillance study indicated that the increased risk of MACE with tofacitinib was mainly observed in the sub-populations with a history of atherosclerotic CVD^45^ and those who were aged ≥65 years or who smoked.^46^ On the other hand, data from STAR-RA,^47^ French,^48^ and Hong Kong biologics (tofacitinib)^33^ registries did not indicate increased frequency of MACE when compared with TNFi.^43^ On the subject of VTEs, results are also conflicting. In ORAL Surveillance, the risk of DVT and PE were comparable between tofacitinib 5 mg recipients and TNFi-treated individuals, but VTE risk (mostly PE) was increased for the 10 mg BD tofacitinib dose,^44^ again mainly in those aged ≥65 years or who smoked.^46^ Data from the Swedish Registry reported a hazard ratio of 1.73 (1.24 to 2.42) for VTE in JAKi-treated RA patients compared to anti-TNFs,^49^ whereas real world data also suggested that there is an augmented risk for VTEs in RA patients treated with baricitinib [IR ratio compared to TNFi: 1.51 (95% CI 1.10, 2.08)].^49^ Similarly, to MACE, there were also studies reporting comparable risk for VTEs between JAKi and bDMARDS, such as those deriving from French and Corona registries^43^ and a population-based study in Korean patients with RA treated with JAKi versus TNFi.^50^

These differences in risk may reflect differences in the populations, with ORAL Surveillance enriched for CVD and VTE risk. Finally, a meta-analysis studying the safety profile of JAKi in patients with PsA and psoriasis who had participated in clinical trials, revealed that neither the occurrence of all CV events (RR 1.18, 95% CI 0.43–3.20, p = 0.75) nor thrombosis (RR 0.83, 95% CI 0.11–6.24, p = 0.85) differed statistically between JAKinibs and placebo arms.^35^

In summary, in RA, there currently appears to be a higher risk for CV and thromboembolic events in patients with additional CV risk factors treated with JAKinibs, with data pertaining mainly to tofacitinib and upadacitinib. It remains unclear whether these findings are disease specific or also apply to PsA and axSpA. It should be noted that the latter are relatively younger with a lower systemic inflammatory burden, albeit with a worse metabolic profile, compared to individuals with RA. Even for RA, more data are needed to establish whether there are differences regarding CV safety between tofacitinib, baricitinib and more selective JAKinibs (filgotinib, upadacitinib) or if this is a true class effect associated with JAKi. To-date, most data for selective JAKi are provided by a safety profile analysis for upadacitinib across RA, PsA, Ankylosing Spondylitis and atopic dermatitis.^51^ Although VTE events were reported in upadacitinib-treated individuals across the aforementioned diseases, the rates were comparable to those described with adalimumab in RA and PsA and MTX in RA.^51^ The possible correlation between JAKinibs and CVD or VTE risk therefore requires further investigation across both diseases and JAKi types.

## CONCLUSION

Selective Janus kinase Inhibitors are a promising therapeutic option and an important ‘arrow in the quiver’ for the clinical physician in the treatment of SpA. Their efficacy for many signs and symptoms of PsA and axSpA, compared to already existing treatments, such as biologic DMARDs, and their satisfactory safety profile when it comes to common comorbidities and infection side effects, is promising regarding their further use in the everyday clinical practice. However, uncertainty remains regarding CVD safety and dedicated PsA- and axSpA-focused, long-term, and larger clinical trials of selective JAKi are required, in order to improve our understanding as doctors as to how best to use these agents to improve the quality of life of individuals living with SpA.
